# Grandparent caregiving in Cambodian skip‐generation households: Roles and impact on child nutrition

**DOI:** 10.1111/mcn.13169

**Published:** 2021-07-09

**Authors:** Mira Leonie Schneiders, Maly Phou, Vira Tun, Maureen Kelley, Michael Parker, Claudia Turner

**Affiliations:** ^1^ Ethox Centre, Big Data Institute, Nuffield Department of Population Health University of Oxford Oxford UK; ^2^ Mahidol Oxford Tropical Medicine Research Unit (MORU), Faculty of Tropical Medicine Mahidol University Bangkok Thailand; ^3^ Centre for Tropical Medicine and Global Health, Nuffield Department of Medicine Research Building University of Oxford Oxford UK; ^4^ FHI 360 Cambodia Office Phnom Penh Cambodia; ^5^ HelpAge Cambodia Battambang Cambodia; ^6^ Angkor Hospital for Children Siem Reap Cambodia; ^7^ Cambodia Oxford Medical Research Unit Angkor Hospital for Children Siem Reap Cambodia

**Keywords:** ageing, care‐giving, family influences, grandparent caregiving, health policy, infant and child nutrition, poverty, qualitative methods

## Abstract

This study aims to understand nutrition‐related roles, responsibilities and ethical issues of grandparents caring for their grandchildren in skip‐generation households in rural Cambodia. Over the past decade, Cambodia has experienced a rise in economic migration of working age populations. This has resulted in increasing numbers of ‘skip‐generation’ households, in which grandparents and grandchildren co‐reside without parents, reflecting potential household vulnerability. This qualitative study involved in‐depth interviews and focus group discussions with Cambodian grandparents who were primary caregivers to grandchildren for six months or longer. A total of 39 grandparents were recruited at two sites in north‐west Cambodia. Interviews and focus group discussions were conducted in Khmer and were recorded, transcribed and translated into English. Data were analysed using thematic analysis. Grandparents in this study looked after an average of three children, aged between two months and 18 years old. Overall, 40% were sole caregivers. Analysis showed that grandparents, particularly grandmothers, played a central role in their grandchildren's health and nutrition. Although grandchildren's health and nutrition were a major priority to grandparents, they reported facing significant challenges to safeguard their grandchildren's and their own nutritional needs. As a result, grandparents frequently faced difficult ethical trade‐offs and prioritised their grandchildren's health and nutrition over their own. This study highlights that in order to improve child nutrition, policies and interventions need to be designed in ways that support and enable grandparent caregivers to meet their grandchildren's health and nutritional needs without neglecting their own.

**Key messages**

- Cambodian skip‐generation grandparents in this study play an essential role in their grandchildren's nutrition, health and development.
- Age‐ and gender‐specific roles mean that grandmothers shoulder the majority of caregiving work within skip‐generation households and often face a double burden of domestic caregiving work and productive labour.
- In a context of poverty and intersecting vulnerabilities, skip‐generation grandparents face significant challenges, barriers and burdens while trying to support grandchildren's everyday care and nutrition, with many struggling to meet their grandchildren's needs.
- As a result of their efforts to balance caregiving responsibilities with securing a livelihood, grandparents frequently face difficult ethical trade‐offs and moral dilemmas.
- The critical role of skip‐generation grandparents demands greater recognition and attention so that programmes and policies can be developed to support this influential group of caregivers.

## 1 INTRODUCTION

New‐born and childhood malnutrition significantly impacts cognitive development and has important implications on children's developmental potential and long‐term trajectory (Black et al., 2016). Although it is commonly assumed that parents—most notably mothers—are the custodians of child health and nutrition, the nutritional status of infants and young children in low‐ and middle‐income countries (LMICs) is greatly influenced by broader familial and household structures (Acharya et al., 2004; Berman et al., 1994).

The ‘household production of health’ (HHPH), a ‘conceptual framework for analysis of health status and health change’, has been influential in advancing the idea that health‐related decision‐making, behaviours and outcomes are largely determined by the role of the household and family and that technical solutions offered through healthcare facilities are insufficient for improving health in LMICs (Berman et al., 1994). Drawing on an ecological perspective of health (Bronfenbrenner, 1979), the HHPH framework highlights the embeddedness of the household within broader socioeconomic and structural systems, recognising the importance of socio‐cultural, economic, political and demographic contexts, healthcare systems and other conditions in shaping the options available to households for engaging with health‐producing behaviours (Berman et al., 1994). The influence of gender‐ and age‐specific roles of household members underpinning the production of health and nutrition have been widely recognised (Aubel & Rychtarik, 2015).

However, despite increasing recognition of the HHPH, relatively little research, policy and programmes have explicitly focused on addressing the intra‐household context and the roles of non‐parental family members in influencing infant and child nutrition. Particularly the roles and contributions of older household members, such as grandparents have remained largely invisible (World Health Organization [WHO], 2015).

Public discourse on population ageing has commonly portrayed older people as burdens on society (Officer et al., 2016; WHO, 2015), with this perception being especially prevalent in LMICs (Officer et al., 2020). However, in reality, older people—particularly in LMICs—continue to make important contributions to their households, societies and economies, including through income support, farming, domestic work and as carers for grandchildren (Aboderin & Beard, 2015; Hong, 2015). These contributions have the potential to benefit the entire household, as grandchildren receive more care, supervision and support, whereas parents are freed up to work for income.

A comprehensive review on the involvement and influence of grandmothers on maternal and child nutrition practices, including studies from 35 countries across Africa, Asia and Latin America, revealed that (i) grandmothers play instrumental roles as advisors to younger women and as caregivers to (grand)children, particularly during pregnancy and breastfeeding; (ii) collectively, grandmother social networks are influential in shaping attitudes and practices related to maternal and child nutrition; and (iii) men's respective involvement is limited (Aubel, 2012).

Cambodia represents a particularly interesting case study for a better understanding of the role played by grandparents in child health and nutrition. Traditionally, multi‐generational living is the norm in Cambodia, with 80% of older people living with their adult children (Knodel et al., 2005). Although grandparents in Cambodia—particularly grandmothers—have historically supported their adult children in caring for their children, this role was previously largely restricted to short periods of daytime caregiving (Kato, 2000). More recently, however, Cambodia finds itself in a unique period of socio‐demographic transition, with growing rates of economic, rural–urban or cross‐border migration of working age populations. This has resulted in an increase in so‐called *skip‐generation* households (MOSVY, 2004; Zimmer & Khim, 2013), in which grandparents and grandchildren co‐reside, without the parents of the grandchild.

Skip‐generation households are not unique to Cambodia and are prevalent in many other parts of the world, especially in LMICs (Das & Zimmer, 2015). They are usually brought about by labour migration (e.g. in South‐east Asia: Ingersoll‐Dayton et al., 2017; Knodel & Nguyen, 2015; and China: Chen et al., 2011, Cong & Silverstein, 2012), death of parents (e.g. in the context of HIV/AIDS in Sub‐Saharan Africa: Zimmer, 2009) or family crisis (e.g. Shakya et al., 2012). Within the context of migration, skip‐generation grandparent caregivers in China have been described as ‘family maximisers’, because of their important role in enabling their adult children's migration by filling the ‘care‐gap’ for grandchildren (Baker et al., 2012). Yet inadequate social support networks have long been identified as a key problem for grandparent caregivers (Hayslip & Kaminski, 2005; McCallion et al., 2000), and many grandparents report caregiver burden, especially when their own or their grandchildren's health is poor (Dowdell, 2004).

Although precise prevalence data on skip‐generation households from Cambodia is unavailable, with an estimated 35% of the population being migrants (International Organization for Migration, n.d.) and 80% of ‘left‐behind’ children estimated to live with their grandparents (Ministry of Planning, 2012), it is clear that Cambodian grandparents play an increasingly significant and long‐term role in providing care to grandchildren. Without grandparents to fill the ‘care‐gap’, children in migrant households may be at risk of abandonment, foster care or dropping out of school, with research showing that Cambodian children who join their migrant parents struggle to access education in their destination due to administrative and financial barriers (Creamer et al., 2017). Despite this reality, Cambodian skip‐generation grandparents have received limited policy, programmatic and research attention.

Existing evidence from Cambodia on the health and nutritional status of grandchildren growing up with their grandparents paints a mixed picture. A study by Vutha et al., considering the impact of migration of adult household members on children's wellbeing, health and nutrition, found that migrant children were more likely to experience illness and injury, and negative long‐term health and nutritional effects, including underweight and wasting (Vutha et al., 2014). However, data were not disaggregated by the adult migrating or the family member(s) staying behind (e.g. parent, sibling or grandparent), thereby limiting conclusions. A qualitative study investigating beliefs and practices during pregnancy, post‐partum and in early infancy found that grandmothers, alongside parents, were important stakeholders in decision‐making related to pregnancy and infant health and that mothers sought advice from the older generation, particularly their mothers (Turner et al., 2017). Another study by Hong, comparing *nuclear* and *multi‐generational* households (i.e. co‐residing grandparents) found improved outcomes for time invested in childcare, nutritional status and risk of infant mortality among multi‐generational households (Hong, 2015). Although the study concluded that the presence of co‐residing grandparents safeguards child health and wellbeing (Hong, 2015), the role of *skip‐generation* grandparents in child health and nutrition, and the specific challenges that grandparents face in this context, presents an important gap in current research, which this study seeks to address.

## 2 METHODS

### 2.1 Study design and aims

The aim of this study was to examine the lived experiences of grandparents caring for their grandchildren in rural, migrant skip‐generation households in north‐western Cambodia, in order to understand grandparents' caregiving roles and responsibilities, the impact on child health and nutrition and ethical issues arising in this context. The study was conducted in collaboration with two Cambodian partner organisations; HelpAge Cambodia in Battambang and Angkor Hospital for Children (AHC) in Siem Reap. Ethical approval was granted.

This study used in‐depth interviews and focus group discussions (FGDs) as complementary methods. First, FGDs were conducted to better understand social norms and gain a range of perspectives on grandparent caregiving, as group discussions are particularly useful for surfacing a breadth and range of experiences and viewpoints (Kitzinger, 2006). Subsequently, in‐depth interviews were conducted to gain a more holistic and nuanced understanding of the individual beliefs and experiences underpinning grandparent caregiving, as individual interviews are well suited for building up a rich and comprehensive picture of lived realities (Herbert & Rubin, 1995). While topic guides for FGDs and in‐depth interviews focused on the same broad themes, interview guides included additional in‐depth questions on individual experiences.

### 2.2 Population and sampling

Grandparents of any age, who self‐identified as primary caregivers to at least one grandchild for at least six months, consecutively or continuously, within the past two years were included in this study. Among those eligible, a maximum variation sample was selected to capture grandparent diversity with regards to age, sex, marital status and the number and age of grandchildren cared for. Participants were recruited through the networks of the two collaborating partners using purposive sampling. Staff in both partner organisations shortlisted grandparents in the NGO outreach programmes and those presenting at the hospital with their grandchildren during the recruitment period.

### 2.3 Qualitative methods and topic guide

All interviews and FGDs were conducted in Khmer by an experienced Cambodian qualitative researcher (Ma P). The lead researcher (M L S) and a translator were present during all interviews and FGDs. FGDs also included a note taker. The topic guide focused on four broad areas: (i) roles, responsibilities and experiences of caregiving; (ii) health of grandchildren; (iii) health of grandparents; (iv) sources of support (topic guide available on request). Interviews and FGDs were semi‐structured, employing open‐ended questioning to support a rich exploration of grandparents lived experience and embedded ethical issues.

### 2.4 Data collection and analysis

Data collection took place in March and November 2018. Eligible participants were contacted via phone or approached directly by partner staff to invite them to take part in the study, share relevant information and arrange a convenient time for an interview or FGD. In Battambang, interviews took place in grandparents' homes and FGDs in a central, easily accessible venue. In Siem Reap, interviews and FGDs were conducted in a designated, private room at the hospital. Following an introduction to the study, informed consent was obtained, and key socio‐demographic information was collected. Data were collected iteratively, with emerging insights informing further data collection, and continued until reaching ‘saturation’, a point beyond which the research team judged additional data collection to be unlikely to reveal ‘new information and themes’ (Guest et al., 2006). A total of three FGDs (*n* = 21) and 18 in‐depth interviews were conducted.

All interviews and FGDs were recorded, transcribed verbatim into Khmer and then translated into English. Analysis of data from translated interview transcripts involved various steps, namely, thinking about the data, coding, analysis, testing and confirming findings and lastly writing up findings (Ziebland & McPherson, 2006). Line‐by‐line coding of transcripts was supported using the qualitative data management software NVivo (version 10.2.2). Thematic analysis using an inductive approach was conducted to make sense of the data and identify ‘repeated patterns of meaning’ (Braun & Clarke, 2006). Thematic analysis describes ‘a method for identifying, analysing and reporting patterns (themes) within data’ (Braun & Clarke, 2006), compatible with various theoretical paradigms. Three researchers participated in reviewing and independently coding transcripts, with MLS coding all transcripts and MiP and MK coding a subset of ≈25% of transcripts, selected for diversity. Coding was discussed to facilitate diversity of perspectives on the data, strengthening trustworthiness, rigour and consistency of data analysis (Morse et al., 2002). Inconsistencies in coding were resolved through discussion among the coding team and with the Cambodian research assistant (MaP) until reaching consensus. Although transcripts from FGDs and in‐depth interviews were initially analysed separately, no salient differences among the major emerging themes were found between these two data sources. For the purpose of this analysis, they have thus been treated as one data set.

### 2.5 Ethical considerations

Ethical approval for this study was granted by Oxford Tropical Research Ethics Committee (OxTREC Ref: 536‐17), the Angkor Hospital for Children Institutional Review Board (AHC IRB Ref: 0158/18) and the Cambodian National Ethics Committee for Health Research (NECHR Ref: 039‐NECHR).

## 3 RESULTS

### 3.1 Socio‐demographics

A total of 39 grandparent caregivers (32 grandmothers and seven grandfathers) participated in this study, across 18 in‐depth interviews (*n* = 18) and three FGDs (*n* = 21) (see Table 1). On average, grandparents looked after three grandchildren, aged between two months and 18 years old (see Table 2). More than half (25/39) looked after at least one grandchild aged five years or younger. Grandparents had low levels of education (44% no education) and 38% were sole caregivers to their grandchildren.

**TABLE 1:** Number of in‐depth interviews and focus group discussions conducted by study location

Recruitment location	In‐depth interviews (IDI)	Focus group discussions (FGD)
Battambang	10	2 (*n* = 16)^a^
Siem Reap	8	1 (*n* = 5)
Total	18	3 (*n* = 21)

^a^ Group 1: *n* = 10; Group 2: *n* = 6.

**TABLE 2:** Key socio‐demographic data of grandparent participants

In‐depth interviews and focus group discussions			
	Total (*n* = 39)	IDI (*n* = 18)	FGD (*n* = 21)
Grandparent gender	*n* (%)	*n* (%)	*n* (%)
Female	32 (82.1)	15 (83.3)	17 (81.0)
Male	7 (17.9)	3 (16.7)	4 (19.0)
Grandparent age (years)			
Mean (range)	60.8 (42–74)	62.8 (53–73)	59.2 (42–74)
Grandparent education			
No education	17 (43.6)	6 (33.3)	11 (52.4)
Some primary	17 (43.6)	9 (50.0)	8 (38.1)
Some secondary	4 (10.3)	2 (11.1)	2 (9.5)
Above secondary	1 (2.6)	1 (5.3)	0 (0.0)
Grandparent marital status			
Never married	0 (0.0)	0 (0.0)	0 (0.0)
Married/living together	24 (61.5)	10 (55.6)	14 (66.7)
Female	18 (46.2)	6 (33.3)	12 (57.1)
Male	6 (15.4)	4 (22.2)	2 (9.5)
Divorced/separated	4 (10.3)	4 (22.2)	0 (0.0)
Female	4 (10.3)	4 (22.2)	0 (0.0)
Male	0 (0.0)	0 (0.0)	0 (0.0)
Widowed	11 (28.2)	4 (22.2)	7 (33.3)
Female	10 (25.6)	4 (22.2)	6 (28.6)
Male	1 (2.6)	0 (0.0)	1 (4.8)
Number of children alive			
Mean (range)	4.9 (1–12)	4.8 (1–8)	5.0 (1–12)
Number of grandchildren alive			
Mean (range)	8.7 (1–45)	8.4 (1–18)	9.0 (1–45)
Number of grandchildren cared for			
Total	108	55	53
Mean (range)	2.8 (1–14)	3.1 (1–14)	2.5 (1–6)
Average age of grandchildren cared for (years)			
Mean (range)	7.8 (0.2–18)	8.4 (0.2–18)	7.1 (0.3–15)
Gender of grandchildren cared for			
Female	56 (51.8)	28 (50.9)	28 (52.8)
Male	52 (48.1)	27 (49.1)	25 (47.2)

### 3.2 Grandparents' caregiving roles and responsibilities

Grandparents reported a wide range of caregiving responsibilities for their grandchildren, including (1) providing basic personal care; (2) managing and supporting nutrition; (3) supporting and managing health and healthcare; (4) supervising and protecting grandchildren; (5) providing a moral education; (6) facilitating and supporting schooling; and (7) generating income and supporting livelihoods. The results that follow specifically focus on grandparents' roles in providing, managing and supporting grandchild nutrition.

### 3.3 Managing and supporting grandchild nutrition

Asked about their daily caregiving responsibilities, the majority of grandparents described that adequately feeding grandchildren was an essential part of their role. Grandparents reported dedicating substantial resources—both time and money—to obtaining and preparing food, with most cooking two to three meals per day. Those who cared for infant and young children described caring and feeding of young grandchildren as particularly time and labour intensive. For example, one grandmother who looked after her two‐year‐old grandson explained:

I get up to feed [my grandson] two times every night. […] At 2 am and at 3:30 am, [he] gets up. […] Because when he is hungry, he tosses his legs. […] When he was still a baby of five or six‐month old, he got up three or four times [each night]. (Grandmother, 64 years, Interview)

Many grandparents also said that it was essential to provide their grandchildren with adequate and good quality nutrition to keep them healthy. Some grandparents discussed the link between a diet balanced in nutrients and grandchildren's health. For example, reflecting on what was important in looking after grandchildren day‐to‐day, one grandmother explained:

Preparing a good meal for them. […] I don't let my grandchildren eat by themselves because they only want to eat meat, so I have to feed them. […] I am scared they'll get sick and also scared if they are too thin, so I need to pay attention to their health. […] I always want to take the best care of them so their parents will be at ease […] I am concerned they will not be happy when they see their children being thin. (Grandmother, 60 years, Focus group discussion)

Furthermore, grandparents felt that obtaining safe drinking water for consumption and food preparation was important for preventing illness among grandchildren. Several grandparents, who obtained water from a local well, expressed worries about its quality, particularly when used to prepare porridge for infants. Many grandparents put substantial time, effort and resources into boiling well water to make it safe for consumption, as highlighted by one grandmother looking after her 11‐month‐old grandson:

I boil water [from the well] and leave it so I can get clear water to cook porridge for [my grandson]. […] I leave the boiled water for a while to let all dregs go down at the bottom and use the clear water to cook porridge. I boil the water twice. […] When the porridge is ready, I mix the water with milk. […] When he sleeps, I do his nappy and laundry and cook the porridge … I get up at 3 am to cook the porridge again. […] I don't let him drink milk mixed with left‐over porridge water… (Grandmother, 66 years, Interview)

### 3.4 Gender‐specific caregiving roles

Among most grandparent‐couple households (24/39), the division of caregiving roles appeared to follow traditional gender norms. Grandmothers more commonly reported responsibility for domestic duties, including cooking, feeding, cleaning, doing laundry and providing basic care to grandchildren, whereas grandfathers were responsible for income generation and physically ‘heavy work’ in farming and at home. Some grandfathers also took on peripheral care roles, like taking grandchildren to school. However, despite these gender roles, many grandmothers reported also doing ‘heavy work’:

Well, I also do the heavy work … but when I force myself too much, I would hurt myself. […] [Carrying water from the well], cutting wood, and coal … yes, those are heavy works. That's for men to do. […] [A]s women, we cannot do those works … [W]e do not have enough energy like when we were younger. And when we get older, the only thing we can do is to divide the work and help each other out. (Grandmother, 66 years, Interview)

Furthermore, in most families—particularly those facing serious financial hardship—both grandparents reported participating equally in income generating work. This meant that many grandmothers faced a double burden of domestic caregiving work, as well as productive labour. However, among the poorest households, the division of roles by gender appeared to be less rigid, necessitated by the need for both grandparents to help secure a livelihood, as highlighted by one grandfather's comment:

We both help each other. […] [This month] I am busy at the rice field. […] I let [my wife] take care of [our grandson] now because soon she will go to [another region] so the responsibility will fall on me. […] She will go to work on a farm. […] I will take care of the chickens, ducks, cows and my grandchildren. […] If we do not share [the responsibilities], we cannot do it all. […] When we share the tasks, it's easier for us. (Grandfather, 55 years, Interview)

Consistent with this view, most grandparents felt that having the support of a spouse was helpful for managing caregiving responsibilities. However, 15 of the 39 grandparents interviewed—all of them women—were solely responsible for all aspects of their grandchildren's care, following widowhood, separation or divorce. Among these grandmothers, the lack of spousal support, coupled with older age poverty, lower social status and reductions in physical strength were reported as considerable challenges for caregiving.

### 3.5 Challenges facing grandparents supporting child nutrition

#### 3.5.1 Poverty related challenges

Grandparent caregivers discussed several challenges they faced while supporting their grandchildren's nutrition, with widespread poverty, financial hardship and food insecurity being identified as key barriers. Many grandparents reported that remittances by their adult migrant children were insufficient, unreliable and irregular. As such, despite most grandparents demonstrating clear understanding of the importance of providing grandchildren with clean water and nutrition adequate in quality and quantity, the majority struggled substantially to do so. Many reported occasional or frequent food shortages:

There were days we had enough to eat and there were days we did not. Sometimes, I only had eggs for my grandchildren to eat. (Grandmother, 61 years, Interview)

Consequently, many grandparents described taking on debt to be able to buy food. Some said they were chronically indebted to local shop keepers. For example, to obtain milk for her infant granddaughter, one grandmother who had stopped working for income, relied on her children's remittances to repay local shop keepers:

I saved the money my children gave me. If not, I would take milk first and pay [the shopkeeper] later. […] These days I can always take things first when I don't have money and when my children send me money I will pay [the shopkeeper] back. […] I live my life day by day. (Grandmother, 66 years, Interview)

To reduce food insecurity, the majority of grandparents—who were physically able to do so—reported working to support their household's livelihood. This included income generating work, like being a manual labourer, seller or motorbike taxi driver, and subsistence work, like farming, fishing and rearing livestock. Grandparents noted that such work provided an important source of food or additional income for buying food.

#### 3.5.2 Grandparent specific challenges

Although the above‐mentioned challenges may also be faced by parental caregivers living in similar circumstances of poverty, several barriers discussed appear to be specific to grandparent caregivers. Firstly, some grandmothers looking after infants and toddlers who lacked money to regularly buy infant formula, highlighted the added challenge of not being able to breastfeed—as they had been able to do for their own children. For example, one grandmother described struggling to buy sufficient infant formula for her one‐year‐old granddaughter:

… I gave her [bottled] milk but not much. […] On some days when I did not have money, I would cook [rice] porridge. […] I cooked it until it was thick and mixed it with sugar. […] Then put it in the bottle for her to eat. (Grandmother, 73 years, Interview)

Secondly, many grandparents reported experiencing age‐related declines in energy, functioning and mobility, which made it difficult to fulfil physically strenuous caregiving tasks. Reflecting on how being a grandparent differed from being a parent, one grandmother said: ‘It's more tiring because my arms and legs hurt’ (Grandmother, 67 years, Interview). Several grandparents also felt less able to engage in productive and subsistence work, as compared with when they were younger, because of the physically demanding nature of such work. For example, one grandmother expressed feeling exhausted from trying to juggle her caregiving responsibilities with efforts to support her family's livelihood:

It's very tiring. I feel tired having to take care of them. The kids are small and they cry. […] It's difficult and I am poor. I have to care for the pigs, chicken and ducks and I don't have enough food to feed them. Sometimes, I carry my grandson while feeding [the animals]. […] I carry him with one hand and feed my animals with my other hand. (Grandmother, 58 years, Interview)

Finally, grandparents also frequently reported worrying about being blamed or judged by parents or healthcare providers for being ‘bad’ caregivers. Despite grandparents essentially taking on ‘parenting’ roles, many said they felt accountable and answerable to their children (the grandchild's parents). For example, some worried about being blamed if grandchildren became too thin or ill due to inadequate nutrition:

Grandmother 1 (58 years): I am also worried they don't eat well enough.

Grandmother 2 (74 years): I am always afraid that they'll get sick. […] [Their parents] will blame me. […]

Grandmother 3 (73 years): I am afraid their father will blame me when seeing their kids are thin.

Grandmother 4 (57 years): When they see their kid being thin, they will ask what is wrong with her? […] (Grandmothers and grandfathers, Focus group discussion)

### 3.6 Nutrition‐related moral dilemmas

Resulting from their responsibility to support their grandchildren's care, health and nutrition, grandparents frequently faced difficult ethical decisions and trade‐offs. Many described facing nutrition‐related moral dilemmas—namely, situations which arose when more than one interest was at play and in conflict with one another.

For several grandparents, the need to work to feed their grandchildren created direct tensions and conflicts with other caregiving roles, most notably the supervision of young grandchildren. For example, one grandmother described morally struggling to decide whether to leave her three‐year‐old granddaughter—who suffered from severe physical and mental disabilities—alone at home while going to work:

If talking about difficulties, there are a lot. When I don't have money, I go to wash dishes for others. […] If I don't spare time to work, I will have no money. […] It was very difficult, I had to leave my grandchild because I had to make money to buy her food. […] If I stopped, I would have no money … to buy milk for my grandchild. […] I felt difficult and sad that I had to leave my grandchild at home because I am poor. I have to put up with it. […] I have to close my eyes to it sometimes. (Grandmother, 51 years, Interview)

The above quote illustrates a moral dilemma faced by several grandparents wishing to supervise and protect their grandchildren from harm, while also needing to go out to work to feed their grandchildren. Consequently, several grandparents saw themselves with no choice but to leave grandchildren unsupervised at home while working.

Grandparents also described making inadvertent trade‐offs between trying to meet each grandchild's everyday nutritional needs *versus* the child's healthcare needs. For example, some grandparents reported not attending preventive or follow‐up appointments with chronically ill grandchildren, because they instead needed money to meet everyday nutritional and basic needs of the child. In this way, many grandparents found themselves constantly struggling to balance grandchildren's long‐term, health‐related interests with more immediate needs for food and nourishment.

Furthermore, most grandparents also described frequent tensions between meeting their own nutritional needs and those of their grandchildren. In their daily struggle to secure sufficient food, several deprioritised and sacrificed their own dietary needs:

Grandfather 1 (74 years): When we have money, we don't buy anything to eat but we'd rather keep it for our grandchildren.

Grandmother 1 (69 years): We eat cold rice that was left overnight.

Grandfather 1: We are old so we can endure.

Grandmother 2 (73 years): ‘Bay kdang’ [leftover crispy rice] is ok for us. (Grandmothers and grandfathers, FGD)

The analysis showed that these moral dilemmas were particularly pronounced among single grandparent caregiver households, where a lack of spousal emotional and financial support often further eclipsed opportunities for grandparents' own self‐care.

## 4 DISCUSSION

Based on the analysis of qualitative interviews and focus group discussions, this study has described the roles of skip‐generation grandparents in managing and supporting grandchild nutrition, the gender‐specific dimensions of caregiving, and key challenges and ethical issues arising from these caregiving responsibilities. Grandparents in this study played an essential role in managing and supporting their grandchildren's care, health and nutrition, while also engaging in income generation, and supporting grandchildren's personal care, supervision, moral and formal education—thereby enabling their adult children to migrate for work. This study offers evidence in support of extensive research documenting the influential role of grandmothers as advisors and caregivers on child nutrition in LMICs (Aubel, 2012, 2020).

Despite grandparents placing high priority on their grandchildren's nutrition and demonstrating good understanding of the importance of nutritional health, grandparents faced numerous challenges and structural barriers in safeguarding nutrition in a context of household poverty, intersecting vulnerabilities and food insecurity. Challenges were heightened for grandparents caring for infant and young grandchildren and single grandparents who lacked spousal support. Although similarly positioned parents may also face comparable structural barriers, this study reveals that grandparents experienced additional and unique challenges, including age‐related barriers like declines in energy and functioning (creating challenges for caregiving and income generation) and inability to breastfeed (resulting in additional costs for infant formula). In line with these findings, a comparative study of caregiver burden found that grandparent caregivers experienced worse mental and physical health outcomes than adult caregivers (Strawbridge et al., 1997). Importantly, grandparents are likely to struggle with intensive caregiving responsibilities in their later years—in a life‐phase in which they might reasonably have expected to have more ‘off time’ (Goodman, 2006)—not only because of functional decline but also because they lack access to the same social support, material and practical resources available to parents. Additionally, grandparents in this study also worried about being blamed by parents and health care providers for ‘bad’ grandparenting. Similarly, a study from Thailand found that the responsibilities placed on grandparent caregivers represented a considerable source of worry, partially mediated by difficult relationships between grandparents and their migrant children (Ingersoll‐Dayton et al., 2020).

The majority of grandparent caregivers taking part in this study were women (see Table 2). This reflects both demographic trends in Cambodia—where more women than men survive into older age—as well as customs and gender norms, which make it more likely for grandmothers to take on care of grandchildren (Knodel & Zimmer, 2009; Zimmer & Kim, 2001). Findings from this study show that grandparents' caring roles largely followed gender‐based norms, with grandmothers being predominantly responsible for caregiving and domestic work. Similarly, in many other cultures, gender‐ and age‐specific roles of older women mean that grandmothers are regarded as having acquired expertise and authority in child rearing (Aubel, 2012; Aubel & Rychtarik, 2015). Evidence from multi‐generational households in Cambodia shows that grandmothers spend more time caring for their grandchildren, whereas grandfathers spend more time on market labour (Hong, 2015). However, in this study, in addition to caregiving and domestic work, many grandmothers engaged in income and subsistence labour—thus resulting in a ‘double burden’, which was heightened for single grandmothers who lacked spousal support. Importantly, these findings indicate that these skip‐generation grandmothers shoulder unequal caregiving burdens.

Finally, grandparent caregivers frequently reported facing difficult ethical trade‐offs, including around supervision of grandchildren while going out to work, rationing their own meals to feed grandchildren or choosing between spending money on grandchildren's healthcare or nutrition. These moral dilemmas poignantly highlight how in a skip‐generation context, grandparents' agency to fulfil their caregiving roles is fundamentally constrained by the intersectionality of their generational position (i.e. their age, physical health and functioning, and access to resources), their gendered roles (i.e. grandmothers' ‘double burden’) and wider structural circumstances of their lives (i.e. poverty). Faced with moral dilemmas, many grandparents consequently reported deprioritising and neglecting their own health and needs. This finding echoes other studies showing that grandparent caregivers neglected their own health while caring for their grandchildren (Grinstead et al., 2003).

By offering insights into grandparents' caregiving roles, as well as the structural barriers to supporting grandchildren's nutritional health, this study offers a rich perspective on the production of health within skip‐generation households—a context that has thus far been understudied. The HHPH framework (Berman et al., 1994) highlights the importance of acknowledging and understanding the role of households and family systems in the production of health and nutritional outcomes. Consistent with the conceptual underpinnings of this framework, this study has shown that skip‐generation grandparent heads of household are largely responsible for grandchildren's health, nutrition and healthcare—particularly for infants and young children—including illness prevention, provision of treatment and seeking care. However, in a context of intersecting vulnerabilities, including gendered‐role expectations, age‐related functional decline, and structural barriers like poverty and lack of access to support, grandparents struggle to exercise sufficient agency to fulfil the demands of their caregiving roles. These findings add to a growing evidence base documenting the pivotal role of grandparent caregivers, particularly grandmothers (Aubel, 2012) and the struggles they face (McCallion et al., 2000), and provide data in support of the HHPH framework (Berman et al., 1994; Sacks et al., 2019).

Our findings raise a number of important practical considerations. Firstly, they suggest that strengthening efforts to support grandparent caregivers—especially grandmothers—could support their grandchildren to live healthier lives. The explicit inclusion of grandparent caregivers in maternal and child health and nutrition policies and programmes thus presents an important direction for future interventions. Greater consideration needs to be given to moving beyond a narrow focus exclusively targeting parents or mothers, towards explicit inclusion of alternative caregivers—most notably grandparent caregivers. Secondly, gendered divisions of labour among couple‐grandparent households, and higher proportions of single‐grandmother households suggest that older women shoulder the majority of caregiving burden for their grandchildren in Cambodian skip‐generation households. This means that the inclusion of grandmothers—particularly those who are single—into initiatives to improve maternal and child nutrition demands prioritisation. Finally, the heightened demands of caring for infant and young grandchildren, who may be more vulnerable to malnutrition, warrant additional support for respective grandparent caregivers.

The important, yet largely invisible work of grandparent caregivers demands greater recognition and attention in the development of programmes and policies. This study has offered rich insights that highlight the influential roles, and the complex ethical and practical challenges faced by grandparents supporting grandchild nutrition within rural Cambodian skip‐generation households. In order to develop effective child nutrition and health programmes, the lived realities and socio‐cultural dimensions of grandparent caregiving need to be taken into account. Evidence on effective interventions to support grandparent caregivers exists, albeit primarily stemming from high‐income settings (McLaughlin et al., 2017). NGOs in Cambodia are piloting programmes to address the role of grandmothers in improving maternal and infant feeding practices (‘Grandmother Inclusive Approach’, HelpAge Cambodia and World Vision Cambodia, personal communication) and are providing training on healthy eating—including children's nutrition and BMI—to older people through ‘Older Peoples Associations’ (HelpAge Cambodia, personal communication). Findings and recommendations from this study have been shared with these NGOs to help inform early programme development.

### 4.1 Limitations of this study

As this study included grandparents who were NGO service users (via AHC or HelpAge Cambodia), findings may not be easily generalisable to other skip‐generation grandparents across Cambodia. This is because grandparents recruited for this study are likely to represent more disadvantaged and vulnerable rural families, thus rendering them to seek NGO services. At the same time, grandparents presenting at hospital are likely to reflect those more actively involved in and informed about their grandchildren's healthcare, hence leading them to seek specialist care. Future research in Cambodia should include large scale quantitative surveys to expand upon our findings and ascertain their generalisability. Despite these limitations, this study sheds light on some of the complex issues facing Cambodian skip‐generation grandparents and we hope these findings this will prompt further research in Cambodia and elsewhere.

## 5 CONCLUSION

The extent to which older people are able to contribute to their families, households and communities through the care of grandchildren depends heavily on their health in later years (WHO, 2016). Child health programmes targeting grandparent caregivers therefore need to be designed in ways that are sensitive to the needs of grandparent caregivers, in order not to perpetuate existing intergenerational inequalities, but to enable grandparents to meet their caregiving responsibilities without inadvertently undermining their own nutrition, health and wellbeing.

## CONFLICTS OF INTEREST

The author declare that they have no conflicts of interest.

## CONTRIBUTIONS

MLS designed the research study with input and practical support from MK, MiP, MaP, VT and CT. MLS and MaP conducted the research. VT and CT supported the implementation of the study. MLS analysed the data with support from MK and MiP. MLS wrote the draft manuscript, CT, MK and MiP critically reviewed the manuscript. All authors contributed to the draft paper and approved the final version.

## Data Availability

Research data are not shared.
