# Corrigendum

**DOI:** 10.1111/mcn.13269

**Published:** 2021-09-27

In Schneiders et al., 2021, the surname of the third author was misspelled as “Tun” in the author byline and how to cite section. The author's correct name should be “Vira Tum”.

The online version has been corrected.
